# Comparing Ready‐to‐Use and Powder AbobotulinumtoxinA for Glabellar Lines: A Randomized, Controlled, Triple‐Blinded Clinical Trial

**DOI:** 10.1111/jocd.70308

**Published:** 2025-06-23

**Authors:** Silvio Ventura da Silva Junior, Maria Luiza Boechat Borges Neves, Alfonso Sanchez‐Ayala, Pedro Miguel Teixeira Carvas Cebola, Ana Claudia Carbone, Mariana Barbosa Câmara‐Souza, Rodrigo Lorenzi Poluha, Giancarlo De la Torre Canales

**Affiliations:** ^1^ Department of Dentistry Ingá University Center, Uningá Maringá Paraná Brazil; ^2^ Department of Dentistry University of Ponta Grossa Ponta Grossa Paraná Brazil; ^3^ Egas Moniz Center for Interdisciplinary Research (CiiEM), Egas Moniz School of Health & Science Caparica, Almada Portugal; ^4^ Orofacial Pain Unit CUF Tejo Hospital Lisboa Portugal; ^5^ Department of Dentistry State University of Maringá Maringá Paraná Brazil; ^6^ Orofacial Institute of the Americas – IOA Maringá Maringá Paraná Brazil; ^7^ Division of Oral Diagnostics and Rehabilitation, Department of Dental Medicine Karolinska Institutet Huddinge Sweden

**Keywords:** abobotulinumtoxinA, botulinum toxin type A, glabellar wrinkles, ready‐to‐use abobotulinumtoxinA

## Abstract

**Background:**

Botulinum toxin A (BoNT‐A) is the most used procedure to treat glabellar lines; however, limited data exist about the effectiveness of ready‐to‐use BoNT‐A (RTUaboBoNT‐A) for this indication.

**Aims:**

This study compared the efficacy, durability, and safety of RTUaboBoNT‐A for moderate and severe glabellar wrinkles.

**Methods:**

This randomized triple‐blinded trial included 38 male volunteers aged between 25 and 50 years. Participants were randomly divided into two groups: abobotulinumtoxinA (aboBoNT‐A, *n* = 18) and RTUaboBoNT‐A (*n* = 20). Groups received 10 U for procerus muscle and 20 U for the corrugator muscle. Assessed variables included, electromyography activity (EMG), Merz 5‐point glabellar lines scale, FACE‐Q appraisal for lines between the eyebrows and Visual Analogue Scale for pain intensity. Assessments were performed before and 1, 2, 3, and 4 months after injections. For differences in EMG and satisfaction scores, the two‐way repeated‐measures ANOVA and Bonferroni's post hoc analyses were conducted. Wrinkle severity scores were analyzed with the chi‐squared test.

**Results:**

Inter‐group comparisons revealed no significant differences in EMG scores in all assessed periods for the corrugator supercili (*p* = 0.11) and the procerus muscles (*p* = 0.93); for severity of glabellar lines, no significant differences were also found in all follow‐ups for rest (*p* = 0.737) and contracted position (*p* = 0.390), as well as for satisfaction with the treatments. However, the RTUaboBoNT‐A group presented higher levels of pain intensity during the injection procedure (*p* = 0.01).

**Conclusion:**

The RTUaboBoNT‐A and aboBoNT‐A are comparable in efficacy, durability, and safety.

## 1 Introduction

Glabellar lines significantly influence an individual's self‐perception of beauty and emotional well‐being [1]. One of the most effective treatments for wrinkles in this area is botulinum toxin type A (BoNT‐A) injections, a neurotoxic protein formed by the anaerobic Gram‐positive bacteria *Clostridium botulinum* , with serotype A being the most extensively studied and clinically utilized [2]. According to the 2023 International Survey on Aesthetic/cosmetic Procedures (ISAPS), BoNT‐A is recognized as the most widely used non‐surgical treatment around the globe [3]. Moreover, the safety and efficacy of BoNT‐A products for the treatment of glabellar lines are well established, irrespective of the formulation used [4, 5].

The currently used formulations of BoNT‐A are supplied as either lyophilized or vacuum‐dried powders that need reconstitution with sodium chloride 0.9% prior to injection [6]. However, they present potential challenges in clinical settings, which include the time and effort required for preparation, difficulties associated with the reconstitution process, precision in dosing, and variability among injectors, all of which may affect the aesthetic outcomes [7]. Considering these issues, there is potential for lyophilized or vacuum‐dried formulations to be supplanted by ready‐to‐use liquid formulations, which would simplify the clinical routines for healthcare professionals.

In this context, a ready‐to‐use liquid formulation of abobotulinumtoxinA (RTUaboBoNT‐A, marketed as Alluzience by Ipsen/Galderma, Lausanne, Switzerland) emerges as a notable option. RTUaboBoNT‐A is a liquid solution comprised of histidine, sucrose, polysorbate‐80, sodium chloride, hydrochloric acid, and water for injection. This formulation can be stored at 25°C for up to 12 h (no opening) without undergoing pharmacological alterations and was specifically developed without the use of animal‐ or human‐derived excipients [8]. RTUaboBoNT‐A has been approved for the treatment of moderate to severe glabellar lines and has demonstrated both short‐ and long‐term efficacy and safety, even after multiple administrations [9, 10]. Additionally, higher rates of satisfaction with its aesthetic results have been reported [11]. A recent clinical trial have also highlighted a preference for RTUaboBoNT‐A over powder formulations of BoNT‐A, due to its ease of use [12].

Due to its relatively recent introduction, there are limited randomized clinical trials evaluating the properties of RTUaboBoNT‐A in a clinical setting. Furthermore, the only studies comparing RTUaboBoNT‐A to powder formulations of BoNT‐A are an open‐label, randomized Phase IV study and a randomized double‐blind placebo and active comparator trial. The latter involves its counterpart, abobotulinumtoxinA (aboBoNT‐A), and both studies reported comparable effectiveness and safety in the treatment of glabellar lines between the two formulations [9, 12]. Consequently, there is a necessity to assess RTUaboBoNT‐A effects in randomized clinical trials with rigorous methodological design. Thus, this randomized, controlled, triple‐blind clinical trial aimed to compare the efficacy, durability, and safety of RTUaboBoNT‐A with its counterpart, aboBoNT‐A, in the treatment of moderate to severe glabellar wrinkles.

## 2 Methods

This clinical trial was approved by the Research Ethics Committee of Uningá University (CAAE: 74101523.2.0000.5220). All participants received information about the research aims and procedures and were asked to provide written informed consent to participate in the clinical trial. This randomized, triple‐blind study took place at a private specialized aesthetic clinic between June and December 2024 and followed to the Helsinki Declaration. Data reporting adhered to the CONSORT guidelines.

### 2.1 Participants

The sample comprised Brazilian men aged between 25 and 50 years (which prevents generalization of the results of this study), complaining about glabellar dynamic wrinkles at severity levels II–IV, according to the Merz 5‐point scale [13]. Exclusion criteria encompassed patients that had previously received BoNT‐A injections for any indication and any aesthetic procedure in the upper third of the face. Also, patients with autoimmune and neuromuscular diseases, that received any vaccine within 3 months prior to the study initiation and currently using drugs that act on neuromuscular junctions were excluded.

The sample size calculation was performed using G*Power 3.1.9.2 software (version 3.1.9.2, Kiel, Germany), based on a pilot study that included three participants (*n* = 3) in each group, focusing on the electromyography of the procerus muscle. The following parameters were considered for an ANOVA with repeated measures and within‐between interaction: α error probability = 0.05, power (1−*β* error probability) = 0.8, number of groups = 2, number of measurements = 5, and effect size *f* value = 0.412. The partial *η* ^2^ and nonsphericity correction ϵ values obtained were 0.145 and 0.444, respectively. The total sample size calculated was 36 subjects. Accounting for an anticipated withdrawal or dropout rate of 5%, a total of 38 subjects was included to be distributed among the groups.

### 2.2 Study Protocol

During the study, participants underwent six assessments. At the first visit, participants were screened according to the specified inclusion and exclusion criteria. Those who qualified received detailed information about the study methods and procedures. They were informed that they would receive one injection session of BoNT‐A using one of the tested brands. Crucially, the investigators administering the treatments, evaluating the outcomes, and the participants were all blinded to the brand of BoNT‐A used. At the second visit, baseline data were collected, the randomization process was completed, and BoNT‐A injections were applied. Follow‐up examinations were then conducted at 1, 2, 3, and 4 months post‐injections. During each follow‐up visit, all variables were assessed.

### 2.3 Randomization and Blinding

Randomization was carried out using a computer program (http://www.randomization.com/) in blocks of two patients, managed by a technician not involved in any other aspect of the study. For each patient, the technician placed the assigned BoNT‐A brand into a sealed darkened envelope. The randomization roster remained undisclosed to the investigators until data collection was completed. Consequently, the block size and treatment assignments were unknown to the investigator administering the substances (A.C.C), the investigator evaluating the patients (S.V.S.J.), and to the patients themselves. The envelopes were opened immediately before the injections by a third investigator (M.B.C.‐S.), who was exclusively responsible for preparing the syringes. The syringes were pre‐labeled with the corresponding patient identification code and positioned in the clinical room prior to the entry of both the patient and the researcher responsible for administering the treatment. Both BoNT‐A brands are colorless solutions with an identical appearance (translucent). Blinding success was assessed by questioning the patients at the end of the study about the brand of the BoNT‐A they received. No patient knew the brand that was used in their treatment.

### 2.4 Interventions

The injections protocols were performed according to the published consensus of each brand [14, 15]. The protocol for the aboBoNT‐A group involved reconstituting BoNT‐A vials 500 sU (Dysport, Ipsen, Wrexham, United Kingdom) with 2 mL of 0.9% isotonic sterile saline solution, stored at room temperature, giving a dose of 25 U/0.1 mL. Regarding RTUaboBoNT‐A group (Alluzience, Galderma SA, Lausanne, Switzerland), the solution was provided in a vial as a solution for injection (200 U/mL). Each vial contained 0.625 mL of deliverable volume of solution. The total doses administered in each glabellar region for both groups were 50 U divided into 5 injection points (10 U each). Briefly, the procerus muscle was targeted at a unique injection point on the midline at the level of a line linking the left and right medial canthal ligaments. This injection was performed at a perpendicular angle, maintaining contact with the bone. The corrugator muscles were targeted at four injection points (two on each muscle): a superficial point at the tail region was directed toward the medial and inferior margins of the eyebrows, with an injection angle of 45° relative to the midline and the underlying frontal bone; and a point at the head of the muscle with a 90° angle relative to the midline. No additional injection points were used.

The BoNT‐A brands were prepared by a trained investigator (M.B.C.‐S). Injections were applied by an expert specialist in injectable aesthetic procedures (A.C.C). Injections were done using 1 mL syringes with 27.5G × 6 mm needles.

### 2.5 Outcomes

Outcomes were evaluated at five time points (baseline, 1, 2, 3, 4 months) over the study. As subjective assessments, the Merz 5‐points scale [13], the Face‐Q appraisal of lines between eyebrows [16] and the Visual Analogue Scale [17] (VAS‐assessed just one time) were used. As for objective evaluations, the electromyographic (EMG) recordings of the procerus and corrugator supercilii muscles activity were performed. Muscles electric activity assessed by EMG was the primary outcome. The secondary outcomes were glabellar lines severity (Merz 5‐points scale), satisfaction with the treatment (FACE‐Q lines between eyebrows) and pain intensity (VAS).

#### 2.5.1 Electromyography

Electromyography (EMG) signal was recorded with a four‐channel EMG system (Miotool NG USB, Porto Alegre, RS, Brazil, frequency range: 10–700 Hz; sampling rate 3000/s; resolution: 2.44 V/bit) by a single‐calibrated investigator. Bipolar surface electrodes, size 3.2 × 2.8 cm (Ag‐AgCl disks, Covidien llc, Quebec, Canada), were fixed at the cranial end of the nasal bones and at the midpoint of the cranial border of the eyebrow for procerus and corrugator supercili muscles, respectively [18]. The reference electrode was placed on the manubrium of the sternum. Procerus and corrugator supercili electrical activity was recorded during maximum voluntary contraction. For this, participants were asked to contract the assessed muscles (perform glabellar frowning) as much as possible for 5 s. Participants were instructed to perform the contractions three times, and a 2‐min rest between them was allowed to avoid muscle exhaustion.

To obtain the root mean‐square (RMS) value pertaining to 5 s of MVC of the assessed muscles, the EMG signal was recorded at a frequency of 1000 Hz, followed by band‐pass filtering for 20–500 Hz. MiotecSuite software 1.0 (Miotec Equipamentos Biomedicos, Porto Alegre, Brazil) was employed for EMG signal analysis. For statistical analyses the mean of the three recordings was used.

#### 2.5.2 Severity of Glabellar Lines

The severity of glabellar lines in maximum contracted and rest state was assessed using the Merz 5‐point scale. This scale rates the visible lines as follows: 0 = no lines, 1 = mild lines, 2 = moderate lines, 3 = severe lines, 4 = very severe lines [13]. The rating was based on a visual inspection performed by the patients.

#### 2.5.3 Patient's Perceived Satisfaction With Treatment

In this study, the FACE‐Q appraisal focused on lines between the eyebrows was employed to measure patient satisfaction with the treatment. This scale evaluates the extent to which participants were bothered by the area between their eyebrows over the previous week, using a 4‐point scale where 1, 2, 3, and 4 scores, which means “not at all,” “a little,” “moderately,” “extremely,” respectively. Scoring involves summing the individual item scores to generate a total raw score, which is then converted, using a specific conversion table, into a range from 0 (indicating the worst outcome) to 100 (representing the best outcome) [16].

The authors of this study have secured a license agreement to employ the FACE‐Q scales for nonprofit academic research purposes.

#### 2.5.4 Subjective Pain Intensity

Patients scored their subjective pain intensity related to the treatment injection in the glabellar muscles on a 0–10 cm Visual Analogue Scale (VAS) with the endpoints “no pain” and “worst imaginable pain” [17]. Participants were required to mark on the VAS indicating the level of pain during injections.

#### 2.5.5 Adverse Events

Patients received a telephone call after 2 weeks of treatment injections to report any adverse events caused by the treatments.

#### 2.5.6 Statistical Analysis

The data were analyzed using IBM SPSS Statistics (version 25, IBM: NYSE, Armonk, NY, USA). All inferences were conducted with two‐tailed tests, assuming a significance level of 95% (Type I error, *α* = 0.05) and a statistical power of 80% (Type II error, *β* = 0.2). The assumptions of normality and homogeneity of variances were verified using the Shapiro–Wilk test and Levene's test, respectively. Differences in electromyography (EMG) activity and satisfaction scores based on time and toxin were analyzed using a two‐way repeated measures ANOVA. Post hoc analysis was conducted with Bonferroni's correction for marginal means comparisons. The assumption of sphericity was verified using Mauchly's Test of Sphericity. A Student's *t*‐test was performed to compare age and VAS values between the groups. Meanwhile, wrinkle severity scores were analyzed with the chi‐squared test. The power calculated during the analysis of the variables for our sample of 38 subjects was as follows: 0.803 (partial *η* ^2^ = 0.052; effect size *f* = 0.234; nonsphericity correction *ϵ* = 0.721), 0.800 (partial *η* ^2^ = 0.006; effect size *f* = 0.078; nonsphericity correction *ϵ* = 0.583), 0.802 (partial *η* ^2^ = 0.013; effect size *f* = 0.115; nonsphericity correction *ϵ* = 0.679), 0.801 (effect size *w* = 0.229; degrees of freedom = 4), and 0.802 (effect size *w* = 0.329; degrees of freedom = 4) for the Corrugator supercilii and Procerus EMG activities, Face‐Q satisfaction, and wrinkles at rest and during contraction, respectively.

## 3 Results

### 3.1 Study Population

**FIGURE 1:**
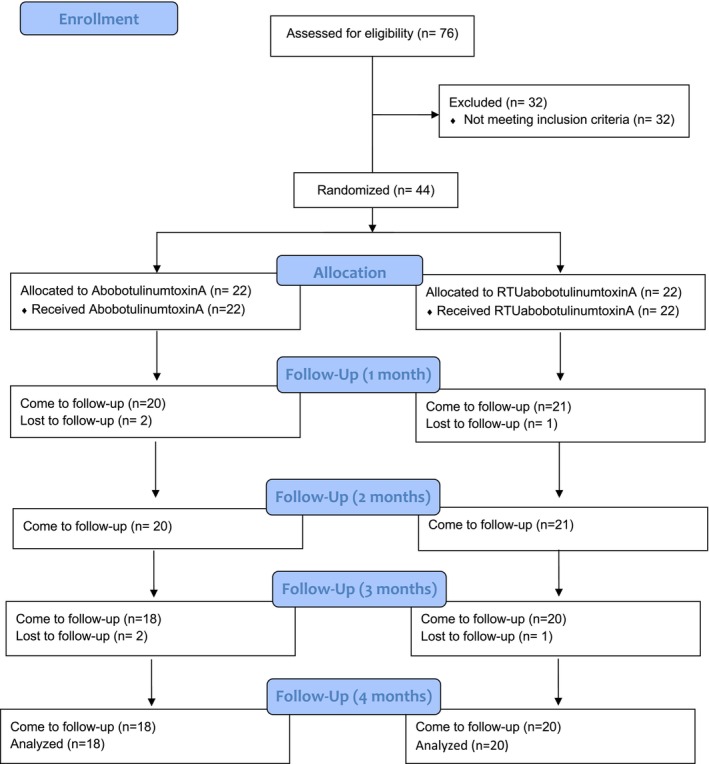
CONSORT flow diagram.

In total, 38 Brazilian men patients underwent screening (32.1 ± 2.3 years old) (aboBoNT‐A: *n* = 18 and RTUaboBoNT‐A: *n* = 20) (Figure 1). No significant differences in age were found between groups (*p* > 0.05) (Table 1). Similarly, no significant differences were identified between groups regarding the distribution of glabellar wrinkles severity in contracted position at baseline (*p* > 0.05) (RTUaboBoNT‐A: 50% (11) very severe, 41% (9) severe, 9% (2) moderate and aboBoNT‐A: 55% (12) very severe, 36% (8) severe, 9% (2) moderate).

**TABLE 1:** Mean and standard deviation (±SD) of age and pain intensity (VAS) compared between groups.

Groups			
	AboBoNT‐A	RTUaboBoNT‐A	*p*
Age	31.7 ± 4.8	31.5 ± 6.4	0.9
VAS	2.5 ± 1.9	4.3 ± 2.1	0.01*

Abbreviations: AboBoNT‐A, abobotulinumtoxinA; RTUaboBoNT‐A, ready‐to‐use abobotulinumtoxinA; VAS, Visual Analogue Scale.

* *p* < 0.05.

### 3.2 Electromyographic Activity

**FIGURE 2:**
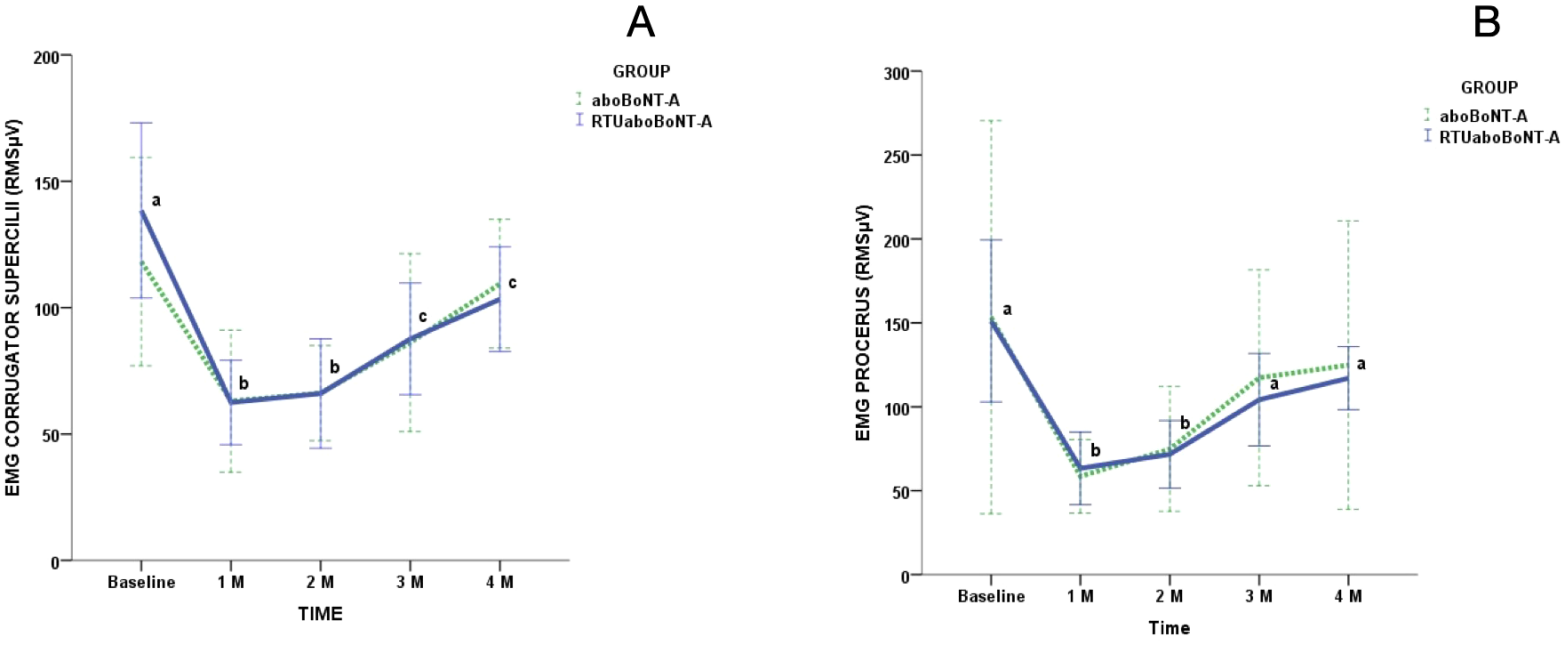
Changes in root mean square scores (RMS μV) in maximum voluntary contraction (MVC) of (A) corrugator supercilli and (B) procerus according to toxin group and timepoints. Different lower‐case letters mean significant intra‐group differences at 0.05. No inter‐group differences were found in the study.

Intra‐group comparisons revealed significant reductions in the electrical activity of the corrugator supercilii at all follow‐up intervals for both groups when compared to baseline (*p* < 0.0001) (Figure 2A). In contrast, both groups exhibited a significant reduction in procerus muscle activity just at the 1‐ and 2‐month follow‐ups compared to baseline (*p* = 0.0001) (Figure 2B). Inter‐group comparisons revealed no significant differences between the groups in all assessed periods for the corrugator supercilii (*p* = 0.117) and the procerus (*p* = 0.935) muscles (Figure 2A,B).

### 3.3 Glabellar Lines Severity

**FIGURE 3:**
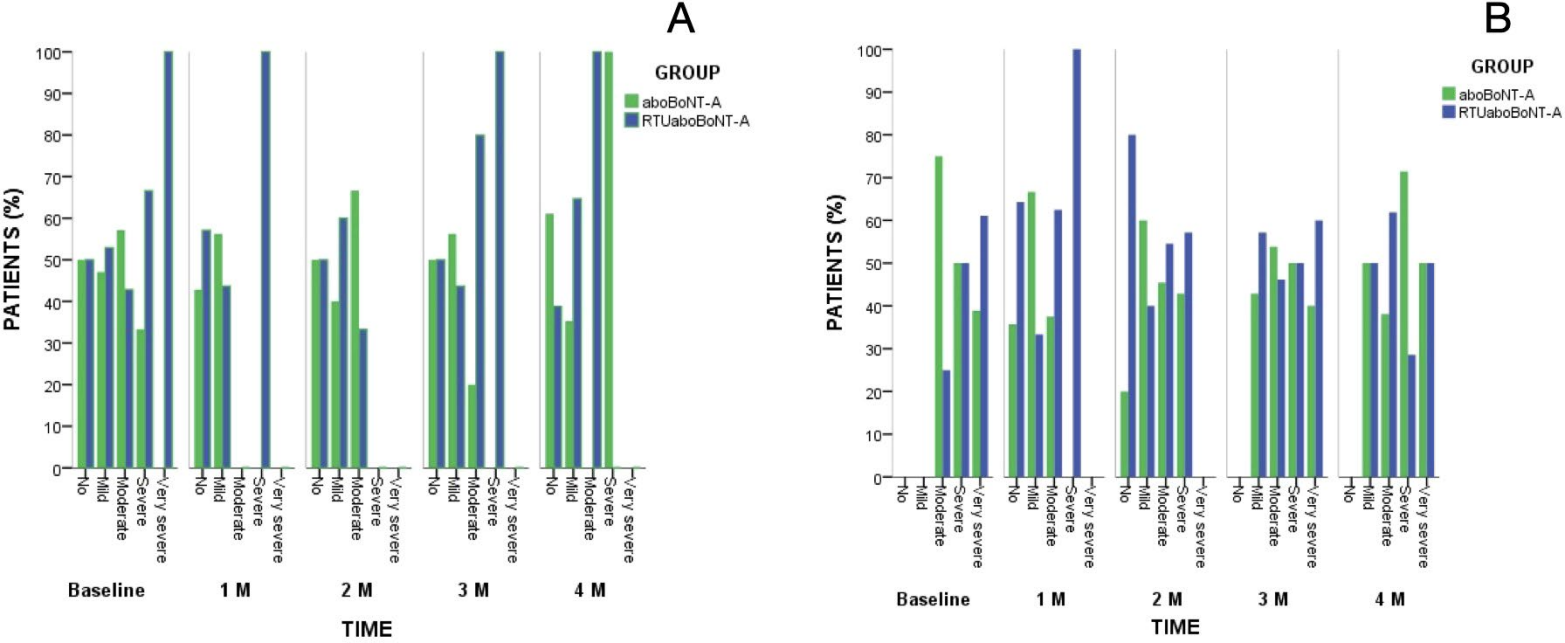
Frequency of Merz scores according to toxin group and timepoints at (A) rest and (B) contracted positions. Significant intra‐group differences at 0.05 were found in both assessed positions. No inter‐group differences were found in the study.

Intra‐group comparisons showed significant reductions in glabellar wrinkles severity for both groups across all follow‐ups when compared to baseline (*p* = 0.001) in the contracted position (Figure 3A). These significant differences were found at the rest position just at the 1‐ and 2‐month follow‐up for both groups (*p* = 0.01) (Figure 3B). Inter‐group comparisons exhibited that the proportion of subjects in each degree of the wrinkle severity scale for each treatment at each time point was similar for both the rest (*p* = 0.737, *χ* ^2^ = 1.996) and contracted (*p* = 0.390, *χ* ^2^ = 4.119) positions with no significant differences between groups (Figure 3A,B).

### 3.4 Patient Satisfaction With Treatment

**FIGURE 4:**
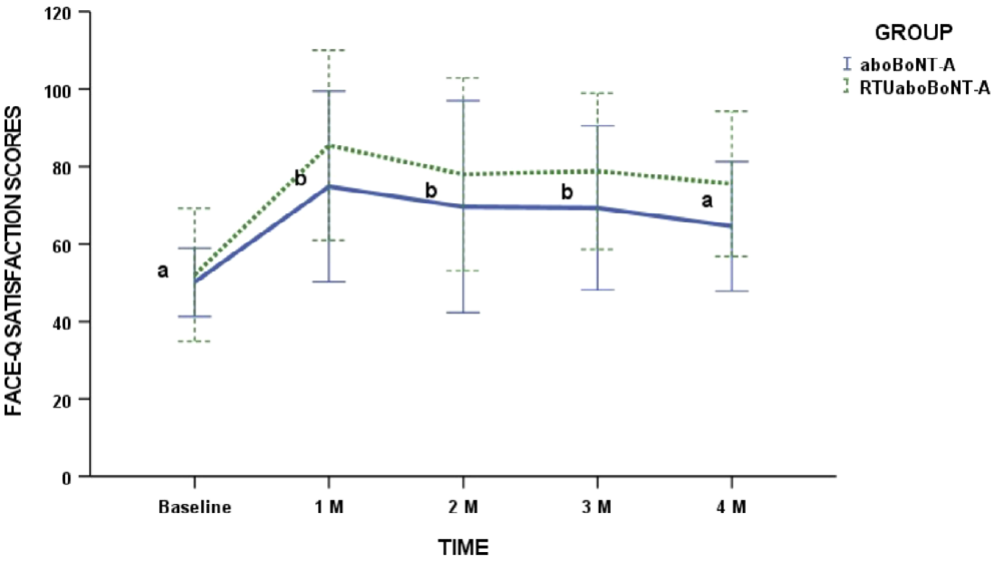
Face‐Q satisfaction scores for each toxin group in different timepoints. Different lower‐case letters mean significant intra‐group differences at 0.05. No inter‐group differences were found in the study.

Regarding intra‐group comparisons, both groups promoted patient's satisfaction with treatment at the 1‐, 2‐, and 3‐month follow‐ups compared with baseline (*p* = 0.0001). Conversely, no significant differences were observed between groups (*p* = 0.766) in all assessed periods (Figure 4).

### 3.5 Pain Intensity

Table 1 presents the mean and standard deviation of VAS scores for pain intensity during treatment injection. It was observed that subjects treated with RTUaboBoNT‐A reported higher levels of pain during the injection procedure (*p* = 0.011).

### 3.6 Adverse Events

No adverse events were reported for any of the participants in both groups throughout the study.

## 4 Discussion

The advent of RTUaboBoNT‐A marks a pivotal advancement in the field of aesthetic medicine. This formulation was the first ready‐to‐use liquid solution introduced in Europe for addressing the severity of glabellar lines [8]. Designed with specific practical benefits to minimize preparation errors, it also assures consistent dosing accuracy and optimizes clinical efficiency [9, 10, 12, 19]. Although these logistical advantages are noteworthy, the RTUaboBoNT‐A retains an identical purified neurotoxin type A complex as its lyophilized powder predecessors, yet it incorporates distinct components and avoids excipients of animal or human origin. These compositional variations could potentially affect clinical outcomes, warranting further investigation.

In this context, questions persisted regarding whether the clinical effectiveness of RTUaboBoNT‐A could rival or exceed that of the powdered formulation, which boasts a well‐established record of efficacy and safety in diminishing the severity of glabellar lines. The present study undertook a comparative analysis of the efficacy, durability, and safety between the RTUaboBoNT‐A and its powdered counterpart aboBoNT‐A. Our investigation revealed that both BoNT‐A formulations successfully decreased muscle electrical activity, ameliorated glabellar line severity, and enhanced patient satisfaction with the treatment. However, no significant superiority was detected between the two BoNT‐A formulations regarding their efficacy, durability, and safety. Notably, the administration of RTUaboBoNT‐A was associated with increased discomfort during injections, which warrants consideration in clinical practice.

Regarding EMG results, this is the first study assessing the effects of RTUaboBoNT‐A using an objective measurement. Both the RTUaboBoNT‐A and the reconstituted powdered aboBoNT‐A exhibited significant, comparable reductions in EMG activity of the procerus and corrugator supercilii muscles throughout the study period. The reduction in EMG activity, irrespective of the BoNT‐A formulation used, aligns with findings from previous research, which demonstrated that BoNT‐A effectively inhibits neuromuscular transmission, thereby reducing muscle contraction and mitigating wrinkle formation [20]. Furthermore, our results corroborate existing literature indicating that the peak reduction in electrical activity following BoNT‐A injections typically occurs within the first month post‐treatment [20, 21]. It is important to note that a sustained reduction in EMG activity was observed exclusively in the corrugator supercilii muscle during the 3‐ and 4‐month follow‐ups for both groups, while no significant long‐term changes were detected in the procerus muscle. This result can be attributed to the higher doses administered to this muscle compared to the procerus. As corrugator muscles exert greater influence on the development of both static and dynamic glabellar wrinkles, clinicians should prioritize higher doses into them. The doses used in our study followed the recommendations of the consensus of both BoNT‐A formulations [14, 15].

In addition, from a clinical and subjective point of view, our EMG results align with prior research using subjective scales to assess the efficacy of both BoNT‐A in reducing muscle contraction [9, 12, 19]. In this direction, our study demonstrated that both formulations showed significant improvements in wrinkle severity at all evaluation points assessed by the Merz 5‐point Wrinkle Severity Scale. These findings corroborate previous studies that highlighted the sustained effects of aboBoNT‐A and RTUaboBoNT‐A in reducing glabellar line severity [9, 12]. Importantly, no significant differences were observed between both formulations, suggesting equivalent efficacy in reducing the perceived wrinkle severity. This corroborates with prior studies, where the RTUaboBoNT‐A formulation demonstrated a high‐rate effectiveness compared to placebo, and equal effectiveness compared to onabotulinumtoxinA and to its powdered counterpart aboBoNT‐A, with response rates exceeding 80% at peak evaluation points [9, 10, 12]. In addition, a Phase III study reported that the response to RTUaboBoNT‐A was consistent even after repeated treatment cycles [19].

Patient satisfaction serves as a pivotal metric for assessing the success of aesthetic procedures. In this study, both RTUaboBoNT‐A and powdered aboBoNT‐A yielded significant enhancements in patient satisfaction, as measured by the FACE‐Q scale, starting at 1 month post‐treatment and extending through the 3‐month follow‐up period. No significant differences were found between the two formulations. Notably, the most pronounced treatment effects on perceived improvements in lines between the eyebrows for both groups related to expressions of anger, the depth of the lines, and frowning. Our findings align with previous studies demonstrating the effectiveness of RTUaboBoNT‐A in promoting high levels of treatment satisfaction and improved self‐perception [10, 12]. These enhancements in the appearance of glabellar lines suggest that RTUaboBoNT‐A effectively addresses the key goal of patients undergoing aesthetic treatment: achieving post‐treatment satisfaction and a greater sense of well‐being [22].

Pain experienced during BoNT‐A injections is noted as one of the primary drawbacks, potentially causing a patient to discontinue treatment [23]. Therefore, this is a variable that should be considered when selecting the brand of BoNT‐A for treatment. The higher pain perception found in our study for the RTUaboBoNT‐A may be attributed to the physicochemical properties of the formulation. RTUaboBoNT‐A contains excipients like histidine, sodium chloride, and water for injection that alter pH and osmolarity, causing greater local irritation [9, 19, 24] and polysorbate‐80 that have been reported to produce skin irritation and redness. Additionally, technical factors, such as injection speed and needle gauge, could also contribute to the pain experience [25]. In our study, these variables were controlled, as the injections were performed by an experienced specialist in injectable facial aesthetic procedures and new needles were used for each muscle. However, individual variables like tissue sensitivity and pain threshold could have also contributed to the higher percentage of pain shown in the RTUaboBoNT‐A group. These findings underscore the multifactorial nature of injection pain and the need to optimize both formulations and techniques and to use methods for pain reduction to enhance patient comfort during treatment.

The findings of this study indicate that the RTUaboBoNT‐A holds significant clinical implications. By demonstrating efficacy and safety comparable to the powdered formulation, RTUaboBoNT‐A offers practical benefits, including eliminating reconstitution errors, reducing dosing variability, and saving preparation time. Moreover, in related research, RTUaboBoNT‐A has received higher ratings in practical aspects such as comfort and time efficiency. This suggests that its ease of use may enhance the overall treatment experience, despite similar clinical efficacy between the formulations [12]. Collectively, these findings imply that RTUaboBoNT‐A could streamline workflows, especially in high‐demand settings, without compromising treatment outcomes. However, factors like costs and increased pain while injecting should be highlighted before considering RTUaboBoNT‐A as a first option compared with other formulations.

While the study boasts a robust methodological design, certain limitations should be acknowledged. The exclusive inclusion of male participants may limit to extrapolate the findings to female populations, as different doses maybe used in women influencing the durability of the treatments. Furthermore, the study did not assess cost‐effectiveness, a critical factor for the practical adoption of RTU formulations in routine clinical settings and that could impact clinicians' acceptance, accessibility and utilization in aesthetic practices. The 4 months follow‐up could be seen as a short period, however, as a positive patient satisfaction with the treatment were found just until the 3 months of treatment, we decided to finish the study at 4 months after treatment. In addition, our findings differ from previous studies reporting long‐term effects (up to 6 months) of RTUaboBoNT‐A on glabellar severity wrinkles and patients' satisfaction with the treatment [9, 11]. Despite its limitations, the study is distinguished by several notable strengths, including a rigorous design characterized by randomization, triple blinding, and a combination of both objective and subjective assessments. These methodological rigor aspects underscore the study's significant contribution to advancing the understanding of the clinical performance of RTUaboBoNT‐A. We recommend that future research endeavors explore the efficacy of RTUaboBoNT‐A in comparison with other brands and within diverse population groups to further enrich the current knowledge base.

## 5 Conclusion

The study concludes the RTUaboBoNT‐A and aboBoNT‐A are comparable in efficacy, durability, and safety, resulting in favorable clinical outcomes. Given its ease of handling and preparation, RTUaboBoNT‐A emerges as a promising first‐line option for clinicians; however, its increased pain during injections and cost implications should be considered in clinical practice.

## Author Contributions

Conceptualization: S.V.S.J. and G.D.T.C.; data curation: M.L.B.B.N. and S.V.S.J.; formal analysis: G.D.T.C. and A.S.‐A.; investigation: M.L.B.B.N., S.V.S.J., and G.D.T.C.; methodology: S.V.S.J., A.S.‐A., M.B.C.‐S., and G.D.T.C.; project administration: G.D.T.C., M.B.C.‐S., R.L.P., and P.M.T.C.C.; writing – original draft: S.V.S.J., R.L.P., G.D.T.C., A.C.C., and A.S.‐A.: writing – review and editing: S.V.S.J., P.M.T.C.C., A.C.C., and G.D.T.C. All authors have read and agreed with the final version of the manuscript.

## Ethics Statement

This research received approval from the Research Ethics Committee of Uningá University (CAAE: 74101523.2.0000.5220). All participants were asked to provide a signed written consent to participate in the study.

## Conflicts of Interest

Ana Claudia Carbone works as a speaker for Galderma—Brazil. All the other authors declare that they have no competing interests.

## Data Availability

All data are available upon reasonable request to the corresponding author.

## References

[1] A. Carruthers , J. Carruthers , and J. Cohen , “Dilution Volume of Botulinum Toxin Type A for the Treatment of Glabellar Rhytides: Does It Matter?,” Dermatologic Surgery 33 (1 Spec No) (2007): S97–S104, 10.1111/j.1524-4725.2006.32339.x. 17241422

[2] I. Matak and Z. Lacković , “Botulinum Neurotoxin Type A: Actions Beyond SNAP‐25?,” Toxicology 335 (2015): 79–84, 10.1016/j.tox.2015.07.003. 26169827

[3] “ISAPS International Survey on Esthetic/Cosmetic Procedures Performed in 2023,” accessed January 17, 2025, https://www.isaps.org/media/rxnfqibn/isaps‐global‐survey_2023.pdf.

[4] E. Rahman , A. Mosahebi , J. D. A. Carruthers , and A. Carruthers , “The Efficacy and Duration of Onabotulinum Toxin A in Improving Upper Facial Expression Lines With 64‐Unit Dose Optimization: A Systematic Review and Meta‐Analysis With Trial Sequential Analysis of the Randomized Controlled Trials,” Aesthetic Surgery Journal 43 (2023): 215–229, 10.1093/asj/sjac253. 36099476

[5] X. Han , J. Bai , and J. Kuang , “Efficacy and Safety of AbobotulinumtoxinA for Treatment of Moderate‐to‐Severe Glabellar Lines: A Meta‐Analysis,” Ophthalmic Plastic & Reconstructive Surgery 40, no. 2 (2024): 126–133, 10.1097/iop.0000000000002491. 38319153

[6] T. J. Walker and S. H. Dayan , “Comparison and Overview of Currently Available Neurotoxins,” Journal of Clinical and Aesthetic Dermatology 7, no. 2 (2014): 31–39. PMC3935649 24587850

[7] J. Bass Kaplan , “The Dilution Confusion: Easy Dosing for Botulinum Toxins,” Plastic Surgical Nursing 36, no. 1 (2016): 24–27, 10.1097/psn.0000000000000121. 26933983

[8] Alluzience , “Alluzience, 200 Speywood Units/ml, Solution for Injection – ENG SmPC,” accessed January 5, 2025, https://www.lakemedelsverket.se/sv/sok‐lakemedelsfakta/lakemedel?id=20191122000079.

[9] B. Ascher , P. Kestemont , D. Boineau , et al., “Liquid Formulation of AbobotulinumtoxinA Exhibits a Favorable Efficacy and Safety Profile in Moderate to Severe Glabellar Lines: A Randomized, Double‐Blind, Placebo‐ and Active Comparator‐Controlled Trial,” Aesthetic Surgery Journal 38, no. 2 (2018): 183–191, 10.1093/asj/sjw272. 28200002

[10] B. Ascher , B. Rzany , P. Kestemont , et al., “Liquid Formulation of AbobotulinumtoxinA: A 6‐Month, Phase 3, Double‐Blind, Randomized, Placebo‐Controlled Study of a Single Treatment, Ready‐to‐Use Toxin for Moderate‐to‐Severe Glabellar Lines,” Aesthetic Surgery Journal 40, no. 1 (2020): 93–104, 10.1093/asj/sjz003. 30893430 PMC6923737

[11] B. Ascher , B. Rzany , P. Kestemont , et al., “Significantly Increased Patient Satisfaction Following Liquid Formulation AbobotulinumtoxinA Treatment in Glabellar Lines: FACE‐Q Outcomes From a Phase 3 Clinical Trial,” Aesthetic Surgery Journal 40, no. 9 (2020): 1000–1008, 10.1093/asj/sjz248. 31550352 PMC7427150

[12] P. Chadha , P. A. Gerber , S. Hilton , et al., “Ready‐to‐Use AbobotulinumtoxinA Solution Versus Powder BotulinumtoxinA for Treatment of Glabellar Lines: Investigators' and Subjects' Experience in a Phase IV Study,” Journal of Cosmetic Dermatology 23, no. 9 (2024): 2857–2866, 10.1111/jocd.16359. 38807515

[13] A. Carruthers and J. Carruthers , “A Validated Facial Grading Scale: The Future of Facial Ageing Measurement Tools?,” Journal of Cosmetic and Laser Therapy 12, no. 5 (2010): 235–241, 10.3109/14764172.2010.514920. 20825260

[14] B. Ascher , S. Talarico , D. Cassuto , et al., “International Consensus Recommendations on the Aesthetic Usage of Botulinum Toxin Type A (Speywood Unit) – Part I: Upper Facial Wrinkles,” Journal of the European Academy of Dermatology and Venereology 24, no. 11 (2010): 1278–1284, 10.1111/j.1468-3083.2010.03631.x. 20337830

[15] B. Ascher , B. J. Rzany , P. Kestemont , et al., “International Consensus Recommendations on the Aesthetic Usage of Ready‐to‐Use AbobotulinumtoxinA (Alluzience),” Aesthetic Surgery Journal 44, no. 2 (2024): 192–202, 10.1093/asj/sjad222. 37490767 PMC10790960

[16] A. L. Pusic , A. F. Klassen , A. M. Scott , and S. J. Cano , “Development and Psychometric Evaluation of the FACE‐Q Satisfaction With Appearance Scale: A New Patient‐Reported Outcome Instrument for Facial Aesthetics Patients,” Clinics in Plastic Surgery 40, no. 2 (2013): 249–260, 10.1016/j.cps.2012.12.001. 23506765

[17] M. A. Ferreira‐Valente , J. L. Pais‐Ribeiro , and M. P. Jensen , “Validity of Four Pain Intensity Rating Scales,” Pain 152, no. 10 (2011): 2399–2404, 10.1016/j.pain.2011.07.005. 21856077

[18] K. Frank , N. Moellhoff , A. Kaiser , et al., “Signal‐to‐Noise Ratio Calculations to Validate Sensor Positioning for Facial Muscle Assessment Using Noninvasive Facial Electromyography,” Facial Plastic Surgery 37, no. 5 (2021): 614–624, 10.1055/s-0041-1725168. 33682916

[19] P. Kestemont , S. Hilton , B. Andriopoulos , et al., “Long‐Term Efficacy and Safety of Liquid AbobotulinumtoxinA Formulation for Moderate‐to‐Severe Glabellar Lines: A Phase III, Double‐Blind, Randomized, Placebo‐Controlled and Open‐Label Study,” Aesthetic Surgery Journal 42, no. 3 (2022): 301–313, 10.1093/asj/sjab329. 34472596 PMC8844979

[20] M. Alimohammadi , M. Andersson , and A. R. Punga , “Correlation of Botulinum Toxin Dose With Neurophysiological Parameters of Efficacy and Safety in the Glabellar Muscles: A Double‐Blind, Placebo‐Controlled, Randomized Study,” Acta Dermato‐Venereologica 94, no. 1 (2014): 32–37, 10.2340/00015555-1647. 23975053

[21] A. R. Punga , A. Eriksson , and M. Alimohammadi , “Regional Diffusion of Botulinum Toxin in Facial Muscles: A Randomised Double‐Blind Study and a Consideration for Clinical Studies With Split‐Face Design,” Acta Dermato‐Venereologica 95, no. 8 (2015): 948–951, 10.2340/00015555-2093. 25766591

[22] S. E. Cox and J. C. Finn , “Social Implications of Hyperdynamic Facial Lines and Patient Satisfaction Outcomes,” International Ophthalmology Clinics 45, no. 3 (2005): 13–24, 10.1097/01.iio.0000167237.49396.7b. 15970763

[23] S. A. H. Lahrabli , F. Lmidmani , and A. El Fatimi , “Assessment of Pain During the Injection of the Botulinum Toxin: Physical Medicine and Rehabilitation Experience,” Annals of Physical and Rehabilitation Medicine 59 (2016): e148.

[24] M. Alam , A. Geisler , D. Sadhwani , et al., “Effect of Needle Size on Pain Perception in Patients Treated With Botulinum Toxin Type A Injections: A Randomized Clinical Trial,” JAMA Dermatology 151, no. 11 (2015): 1194–1199, 10.1001/jamadermatol.2015.2232. 26352252

[25] R. Strazar and D. Lalonde , “Minimizing Injection Pain in Local Anesthesia,” CMAJ 184, no. 18 (2012): 2016, 10.1503/cmaj.111780. 22546890 PMC3519194

